# Uterus transplant graft's arterial atherosclerotic remodeling veracity

**DOI:** 10.1097/MD.0000000000018612

**Published:** 2020-01-03

**Authors:** Robert Novotny, Jaroslav Chlupac, Jakub Kristek, Jan Pit’ha, Roman Chmel, Eva Sticova, Libor Janousek, Jiri Fronek

**Affiliations:** a Transplant Surgery Department; b Laboratory for Atherosclerosis Research, Institute for Clinical and Experimental Medicine; c Department of Obstetrics and Gynecology, Faculty Hospital Motol; d Department of Pathology, Institute for Clinical and Experimental Medicine; e Department of Anatomy, Second Faculty of Medicine, Charles University, Prague, Czech Republic.

**Keywords:** artery, atherosclerosis, transplantation, uterus

## Abstract

**Background:**

Uterus transplantation is a complex, multi-step experimental procedure used for the treatment of uterus absence or uterus anomaly that prevents embryo implantation or pregnancy completion.

**Method:**

To date, only 51 uterus transplants worldwide had been performed. When simplified, it is vascularized composite allograft transplantation. While it is still an experimental procedure with encouraging results for the future, there are still many issues that have to be clarified. The most serious complications of uterus transplantation are graft rejection or grafts vascular failure.

**Results:**

So far, no reference to the atherosclerotic arterial infiltration of the uterus arteries was suggested and studied as one of the main causes of graft's failure.

**Conclusion:**

In this review we summarized current knowledge and possible role of uterus arterial damage, including atherosclerotic changes on the graft's survival.

## 1 Introduction

Uterus transplantation is a complex, multi-step experimental procedure used for the treatment of uterus absence or uterus anomaly that prevents embryo implantation or pregnancy completion.^[1]^ It affects one per 500 women in childbearing age.^[2]^ The first uterus transplantation was performed by Fageeh et al in 2002.^[3]^ To date, only 51 uterus transplants worldwide had been performed.^[4]^ When simplified, it is a vascularized composite allograft transplantation. While it is still an experimental procedure with encouraging results for the future, there are still many issues that have to be clarified.^[4]^ Currently, the most serious complications of uterus graft rejection or failure have been divided into a peri-procedural or post-procedural. The main peri-procedural complications are lacerations of the veins, arteries, ureter, or bladder wall. The main post-procedural complications are graft rejection, infection, and vascular complications.^[2]^ The main vascular complications are arterial and venous thrombosis. Limited data suggests that the main causes can be; low arterial blood flow, venous outflow problems, or anastomotic blood- flow reduction.^[5]^ So far, no reference to the atherosclerotic arterial infiltration of the uterus arteries was suggested and studied as one of the main causes of graft's failure.

**Figure 1:**
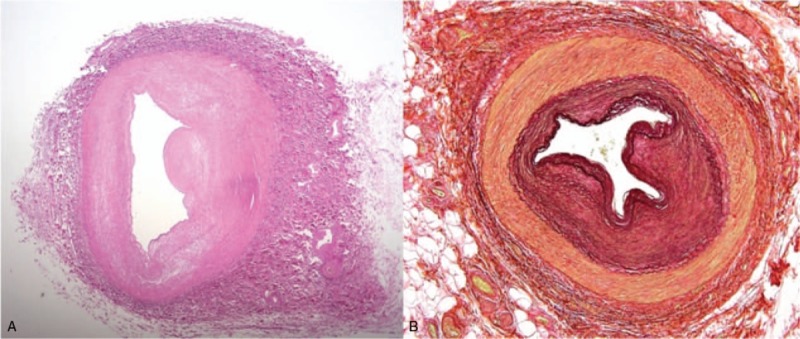
Uterine artery: Cross-section of an artery with circular fibro-intimal thickening of the wall narrowing the arterial lumen. (A) Hematoxilin and eosin, (B), van Gieson and Elastin. Orig. magnification ×40.

Uterus transplantation is a vascularized composite transplantation. Therefore, the status of vasculature in this territory is critical for appropriate function of transplanted uterus grafts (UTx). Vascular complications related to the inflow and outflow vessels could be main culprit for UTx failure (Fig. 1.). Among vascular complications first on the list are arterial and venous thromboses. In addition, Testa et al^[6]^ described also low intimal arterial blood flow, venous outflow problems, and/or anastomotic blood-flow reduction as further causes of UTx failure. However, atherosclerosis, the main reason for arterial and potentially venous damage in general population in most of developed countries was not considered, and studied as a potential etiological factor of UTx failure. In this respect, the status of cardiovascular risk of donors, but also recipients could be of upmost importance.

### 1.1 Estrogen vs atherosclerosis

In addition to 7 main traditional cardiovascular risk factors (age, male gender, family history dyslipidemia, hypertension, smoking, and diabetes mellitus), also hormonal status and its changes are recently intensively discussed. Regarding UTx, significant controversy exists regarding role the of gender specific atherosclerotic changes in smaller arteries (small caliber?). Extremely interesting in this respect is the role of female sex hormones, especially estrogen/estradiol, proved to modulate most of processes in the female vessels including interaction between these vessels and circulating lipids. This effect of sex hormones is considered to be limited only to women.^[6,7,8]^ This was shown by a review conducted by the Society of Thoracic Surgeons Workforce on Evidence-Based Surgery as they demonstrated increasing evidence independent of lipid profile. Postmenopausal women showed a poorer long-term outcome in CABG when compared to the man.^[7,8]^ The primary cause was accelerated atherosclerosis leading to arterial graft failure.^[9]^ The same theory can be applied for UTx arteries. The effect of estrogen on arteries is also very controversial. Some authors associate coronary artery atherosclerotic infiltration with uterine arteries (UA) calcium and plaque formation independent of age and risk factors associated with coronary artery disease.^[10,11]^ Christian et al showed that estrogen might slow down the progression of atherosclerosis in women.^[12]^ Lindner et al demonstrated that peripherally activated estrogen receptor-β seems to be upregulated in vascular endothelium and smooth muscle cells are mediating some of the protective effects of estrogen.^[13]^ We can hypothesize that selective activation of α-estrogen receptors (ER) in a vessel with greater ER density, such as UA, will reduce the post-operative development or progression of vascular atherosclerosis.^[14]^ Liao WX analysis of ER revealed α-ER mRNA is eight times higher than β-ER in the UA endothelium, pointing out a more important role of α-ER vs β-ER. Therefore, based on the above-described insight we can speculate that an artery that is amenable to the estrogenic activation can be an ideal vascular conduit due to the protective effects of estrogen.

### 1.2 Mockeberg atherosclerosis in the uterine artery

Mockeberg atherosclerosis (MocA) is a degenerative disease that involves small and medium-sized arteries.^[15]^ Its main characteristic is that tunica intima remains unaffected and the diameter of the vessel lumen is preserved unlike in common atherosclerosis. Calcium deposits form the middle layer of the wall and the vessels become calcified independent of atherosclerosis.^[16]^ The prevalence of MocA is 13.3% for males and 6.9% for females respectively.^[17,18]^ It can lead to a vascular stiffness reducing the vascular compliance and ability to vasodilate while under increased stress.^[18]^ Amount of data is available on its manifestation on the uterus is however very limited.^[19]^ MocA atherosclerosis does not create arterial stenosis, but the rigidity of the arterial wall can cause in long-term poor UTx perfusion that might result in grafts’ failure.

### 1.3 Fatty acid and cholesterol composition of the uterine artery intima

The concentration of esterified cholesterol, phospholipids and triglycerides increases in the intima-media of arteries with age.^[20]^ During this time the intimal lipid and cholesterol composition nears that of plasma. The fatty acid content of cholesterol esters in the intima of the UA changes over time similarly to the fatty acid pattern of cholesterol esters in plasma low-density lipoprotein. Fatty streak lesions are created, accumulating up to 10 times more cholesterol than the normal arterial intima. In time, this leads to pathological intimal thickening and atheroma formation.^[21]^ Punnonen et al showed a very close correlation between the degree of atherosclerotic infiltration of the UAs, aorta, coronary arteries and carotid arteries.^[22,23]^

The fatty acid chain to cholesterol esters ratio in the intima of the UA is higher than the intima of the aorta.^[20]^ The degree of UA atherosclerosis closely correlates with the risk of cardiovascular disease (increasing age, postmenopausal syndrome, arterial hypertension).^[24]^ Papers by Punnonen R, Jokela H, showed accumulation and increased concentrations of free cholesterol in the intima of the UAs after menopause. The increase of the cholesterol content of the UA intima is not linearly related to the increase of the serum cholesterol concentrations.^[20]^ There is no evidence on the changes in the concentration of long-chain fatty acids in the UAs in pre and post-menopausal period.^[20,25]^ The change of lipid metabolism in the arterial wall in females older than 45 years causes an increase in the intimal concentration of cholesterol esters.^[20]^ This data suggests that women over the age of 45 years may not be an ideal candidate as uterus donors.

### 1.4 Nitric oxide involvement in the pathogenesis of arterial atherosclerosis Decreased nitric oxide (NO) production in endothelial cells is an important pathogenic factor

for atherosclerosis. The accumulation of NO inhibitors (NG-monomethyl-l- arginine, NG- dimethyl-l- arginine) in tissue and plasma explains the decreased NO production.^[26,27]^ NO inhibits platelet adhesion and aggregation which are initiating events for the intimal hyperplasia.^[28]^ The NO inhibitors are associated with diseases such as hypercholesterolemia, intimal hyperplasia, cardiovascular risk factor, or diabetes mellitus.^[2,29,30,31]^

Intimal hyperplasia in UA in premenopausal women is associated with a decrease in endothelium and NO synthases dependent cyclic guanosine monophosphate (cGMP) and the accumulation of NO inhibitors. Furthermore, NO inhibits vascular smooth muscle cells proliferation.^[2]^ The decreased cGMP production servers as a marker for NO productions due to the accumulated endogenous NOS inhibitors and enhanced arginase activity.^[28]^

Therefore, it is up the most importance to manage UTx recipient's hypercholesterolemia, diabetes mellitus and cardiovascular risk factors as they may greatly affect the UA atherosclerotic proliferation, hence they can jeopardize UTx perfusion and clinical performance.

### 1.5 Anatomical variations of the uterine artery

UA origin has many anatomical variations based on angiographic examinations. Up-to-date only few angiographic studies had mapped and classified the origin of UA.^[32,33]^ The latest angiographic study by Albulescu et al in 2014 had confirmed the previously described UA origins; type I (the origin of the uterine artery- inferior gluteal artery ram), type II (the origin of the uterine artery- bifurcation ram inferior gluteal artery), type III (origin uterine artery- the internal iliac artery of the ram trifurcation, with the upper and lower gluteal artery), type IV (proximal to the origin of the uterine artery to the origin of the upper and lower gluteal arteries). However, it disproved the believed occurrence of each type of UA origin, making it more widespread: type I: 24% instead of 45%, type II: 6% instead of 10%, type III: 29% instead of 43%, and type IV: 37% instead of 6%.^[33,34,35]^ Therefore, the most frequent anatomical variations are typy III and IV.

The anatomical variations of UA origin may play a significant role in UTx results especially in deceased donors, where the UA is harvested with the internal iliac artery patch based on anatomical variation. The atherosclerotic infiltration of the arterial patch or in type II and III internal iliac artery can affect the graft's perfusion over time and jeopardies the UTx success.

## 2 Conclusion

Uterus transplantation is a complex, multi-step experimental procedure used for the treatment of uterus absence or uterus anomaly that prevents embryo implantation or pregnancy completion. The protective effects of estrogen on the atherosclerotic arterial infiltration combined with the number of α-ER makes UA an ideal vascular conduit for UTx. The estrogen protective effect is age and in some cases disease dependent. This has to be taken into account when choosing the ideal uterus donor to ensure the best possible grafts function after transplantation.

## Acknowledgment

The author would like to thank Doc. MUDr. Jiri Fronek PhD FRCS and MUDr. Jaroslav Chlupac Ph.D., Transplant Surgery Department, Institute for Clinical and Experimental Medicine, Prague, Czech Republic for providing their opinion on this review.

## Author contributions

**Conceptualization:** Robert Novotny, Jaroslav Chlupac, Jiri Fronek.

**Data curation:** Jakub Kristek, Eva Sticova.

**Formal analysis:** Robert Novotny.

**Investigation:** Roman Chmel.

**Methodology:** Robert Novotny.

**Resources:** Libor Janousek.

**Supervision:** Jiri Fronek.

**Validation:** Jan Pit’ha.

**Writing – original draft:** Robert Novotny, Jan Pit’ha.

**Writing – review & editing:** Robert Novotny, Jaroslav Chlupac, Libor Janousek.

Robert Novotny orcid: 0000-0002-5876-2951.
